# Supplementary material for: Transcriptional profiling of canine osteosarcoma identifies prognostic gene expression signatures with translational value for humans
Source: Commun Biol. 2023 Aug 17;6:856. doi: 10.1038/s42003-023-05208-z (PMC10435536; doi:10.1038/s42003-023-05208-z)
Supplement: Supplementary file 2 — Supplementary Material [file 42003_2023_5208_MOESM2_ESM.pdf]

Supplementary Materials

Supplementary Table 1: Immunohistochemical markers used to identify specific cell types in canine samples

IHC	Cell Type	References
CD20	B cells	[1]
CD204	Macrophages	[2,3]
CD3	T Cells	[4]
CD45ra	Naïve T cells, granulocyte macrophage progenitors	[5,6]
FOXP3	Regulatory T cells	[6,7]
Iba1	Macrophages/Monocytes	[8,9]
MUM1	Subset of germinal B Cells, plasma cells, activated T Cells	[10,11]

Supplementary Table 2: IHC Protocols

Target	Tissue Type	Vendor	Catalog Number	Dilution
CD20	Canine FFPE	Biocare Medical	ACR3004B	1:1000
CD20	Human FFPE	ThermoFisher Scientific/Invitrogen	PA5-16701	1:300
CD204	Canine and Human FFPE	Cosmo Bio	KAL-KT022	1:500 (both)
CD3	Canine and Human FFPE	Agilent Technologies	A0452	1:1000 (canine); 1:100 (human)
CD45ra	Canine FFPE	Peter Moore, UCD	CA21.4B3	1:50
FOXP3	Canine FFPE	ThermoFisher Scientific/Invitrogen	14-5773-82	1:50
Iba1	Canine FFPE	Wako Chemical	019-19741	1:8000
MHCII	Canine FFPE	VMRD	H42A	1:500
MUM1	Canine FFPE	Biocare Medical	CRM352B	1:500

1 Jubala, C. M. *et al.* CD20 expression in normal canine B cells and in canine non-Hodgkin lymphoma. *Vet Pathol* **42**, 468-476, doi:10.1354/vp.42-4-468 (2005).
2 Vázquez, S. *et al.* Immunohistochemical Characterization of Tumor-Associated Macrophages in Canine Lymphomas. *Animals (Basel)* **11**, doi:10.3390/ani11082301 (2021).
3 Seung, B. J. *et al.* CD204-Expressing Tumor-Associated Macrophages Are Associated With Malignant, High-Grade, and Hormone Receptor-Negative Canine Mammary Gland Tumors. *Vet Pathol* **55**, 417-424, doi:10.1177/0300985817750457 (2018).
4 Ferrer, L., Fondevila, D., Rabanal, R., Tarres, J. & Ramis, A. Immunohistochemical detection of CD3 antigen (pan T marker) in canine lymphomas. *J Vet Diagn Invest* **5**, 616-620, doi:10.1177/104063879300500420 (1993).
5 Cimato, T. R., Furlage, R. L., Conway, A. & Wallace, P. K. Simultaneous measurement of human hematopoietic stem and progenitor cells in blood using multicolor flow cytometry. *Cytometry B*

Clin Cytom **90**, 415-423, doi:10.1002/cyto.b.21354 (2016).
6 Boozer, L. B. *et al.* Characterization of immune cell infiltration into canine intracranial meningiomas. *Vet Pathol* **49**, 784-795, doi:10.1177/0300985811417249 (2012).
7 Hori, S., Nomura, T. & Sakaguchi, S. Control of regulatory T cell development by the transcription factor Foxp3. *Science* **299**, 1057-1061, doi:10.1126/science.1079490 (2003).
8 Pierezan, F., Mansell, J., Ambrus, A. & Rodrigues Hoffmann, A. Immunohistochemical expression of ionized calcium binding adapter molecule 1 in cutaneous histiocytic proliferative, neoplastic and inflammatory disorders of dogs and cats. *J Comp Pathol* **151**, 347-351, doi:10.1016/j.jcpa.2014.07.003 (2014).
9 Köhler, C. Allograft inflammatory factor-1/Ionized calcium-binding adapter molecule 1 is specifically expressed by most subpopulations of macrophages and spermatids in testis. *Cell Tissue Res* **330**, 291-302, doi:10.1007/s00441-007-0474-7 (2007).
10 Ramos-Vara, J. A., Miller, M. A. & Valli, V. E. Immunohistochemical detection of multiple myeloma 1/interferon regulatory factor 4 (MUM1/IRF-4) in canine plasmacytoma: comparison with CD79a and CD20. *Vet Pathol* **44**, 875-884, doi:10.1354/vp.44-6-875 (2007).
11 Falini, B. *et al.* A monoclonal antibody (MUM1p) detects expression of the MUM1/IRF4 protein in a subset of germinal center B cells, plasma cells, and activated T cells. *Blood* **95**, 2084-2092, doi:https://doi.org/10.1182/blood.V95.6.2084 (2000).

Supplementary Table 3: ISA gene signatures.

Signature	Genes
ISA 1	A4GALT, ABCA8, ABI3BP, ACKR2, APOA1, ARNTL2, CFD, CFH, CHRDL1, CXCL14, GPRC5A, LSP1, MEDAG, MEOX1, MFAP4, MFAP5, MYH11, NRCAM, PI15, PI16, PRG4, RARRES2, SELP, SEMA3C, SEMA3G, SLC14A1, SLC46A2, THBS4, TMEM176A
ISA 2	ACTA1, CKM, DES, KLHL41, MYBPC1, MYH1, MYH2, MYL1, MYL2, NEB, NRAP, PDK4, PDLIM3, PYGM, RYR1, TMOD4, TNNT1, TNNT3, TXLNB
ISA 3	CD2, CD3E, CD6, CLEC2D, CXCR6, GBP1, GBP5, GIMAP7, GIMAP8, GZMB, GZMK, IL2RB, IL2RG, SECTM1, TBC1D10C
ISA 4	APCDD1L, AQP5, BARX1, BPIFB4, CACNG7, COL11A2, COL2A1, DIPK1C, DLX1, DPYSL5, DUOX1, FADS6, FAT3, FGFR3, GRIA2, GRM4, HORMAD1, HOXB13, HOXC13, IGF2BP1, IHH, IRX1, LHX2, LINGO1, NDNF, NUP62CL, PLPPR2, PPP1R1B, RAB27B, SCML2, SLC13A5, SLC36A2, SLC8A3, SLC9A2, SMPD3, SYT13, TBC1D16, UNC5C, ZIC5

Supplementary Table 4: DOG² Patient Demographics

Number of Dogs	186
Treatment arm	
Standard of Care	93
Standard of Care + Rapamycin	93
Median (range) Age (years)	8.0 (1.4 – 15.6)
Median (range) Weight (kg)	38.6 (25 - 94.5)
Sex	
Castrated Male	100 (54%)
Spayed Female	76 (41%)
Intact Male	7 (4%)
Intact Female	3 (1%)
ALP Status	
Normal	135 (73%)
Elevated	51 (27%)
Tumor Location	
Proximal humerus	39 (21%)
Non-proximal humerus	147 (79%)
Distal Radius	62
Distal Tibia	30
Distal Femur	24
Proximal Tibia	13
Ulna	9
Other	9

*Supplementary Table 5: Summary of DOG^2 clinical outcomes. **Indicates evaluation confirmation by clinical, imaging, and necroscopy evidence*

Median DFI (days)	154
Median Survival (days)	231
Reason off-study	
Disease Progression on Study	83 (44%)
Disease Progression during Follow-up Period	56 (30%)
Complicating Disease / Intercurrent Illness	16 (9%)
Follow-up period completed	11 (6%)
Refused further treatment	9 (5%)
Refused further follow up	3 (2%)
Death on Study	3 (2%)
Death during Follow-Up Period	2 (1%)
Adverse Events / Side Effects	3 (2%)
Dogs dead during the study period	167
Dead with evidence of metastatic disease**	139 (83%)
Dead without evidence of metastatic disease	28 (17%)
Dogs alive at the end of the follow-up period	10
Alive with evidence of metastatic disease	0
Alive without evidence of metastatic disease	10 (100%)
Dogs lost to follow-up	9

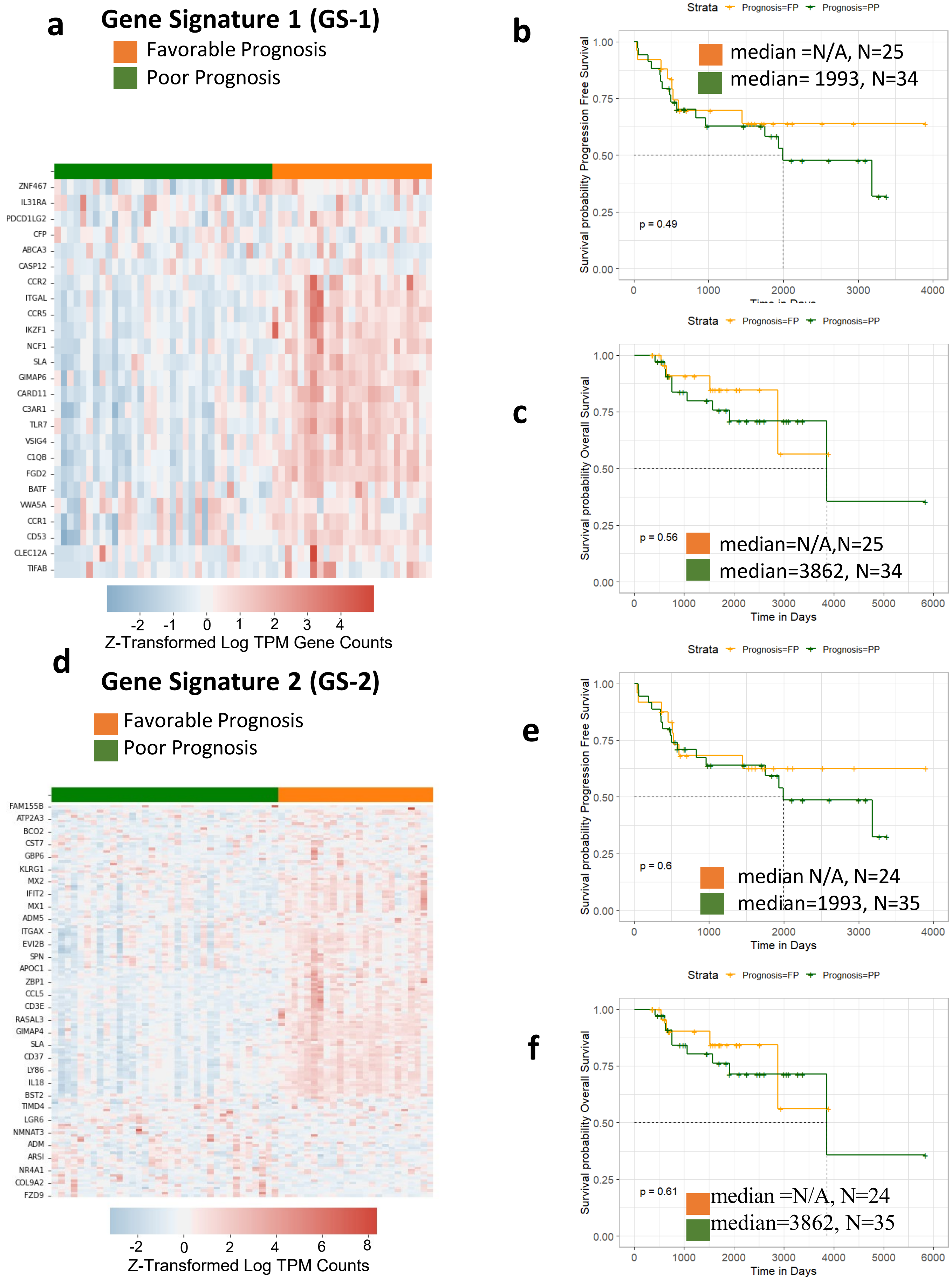

Supplementary Figure 1. Canine Osteosarcoma signature fail to be prognostic in non-metastatic TARGET patients. Despite forming good clusters under GS-1 **a** the Kaplan-Meier curves between clusters is not significant for **b** progression free survival (PFS) or **c** Overall Survival (OSv). Likewise, despite good clustering under GS-2 **d**, Kaplan Meier curves fail to be significantly different for **e** PFS or OSv **f**. Median DFI and Osv are given in days from diagnosis

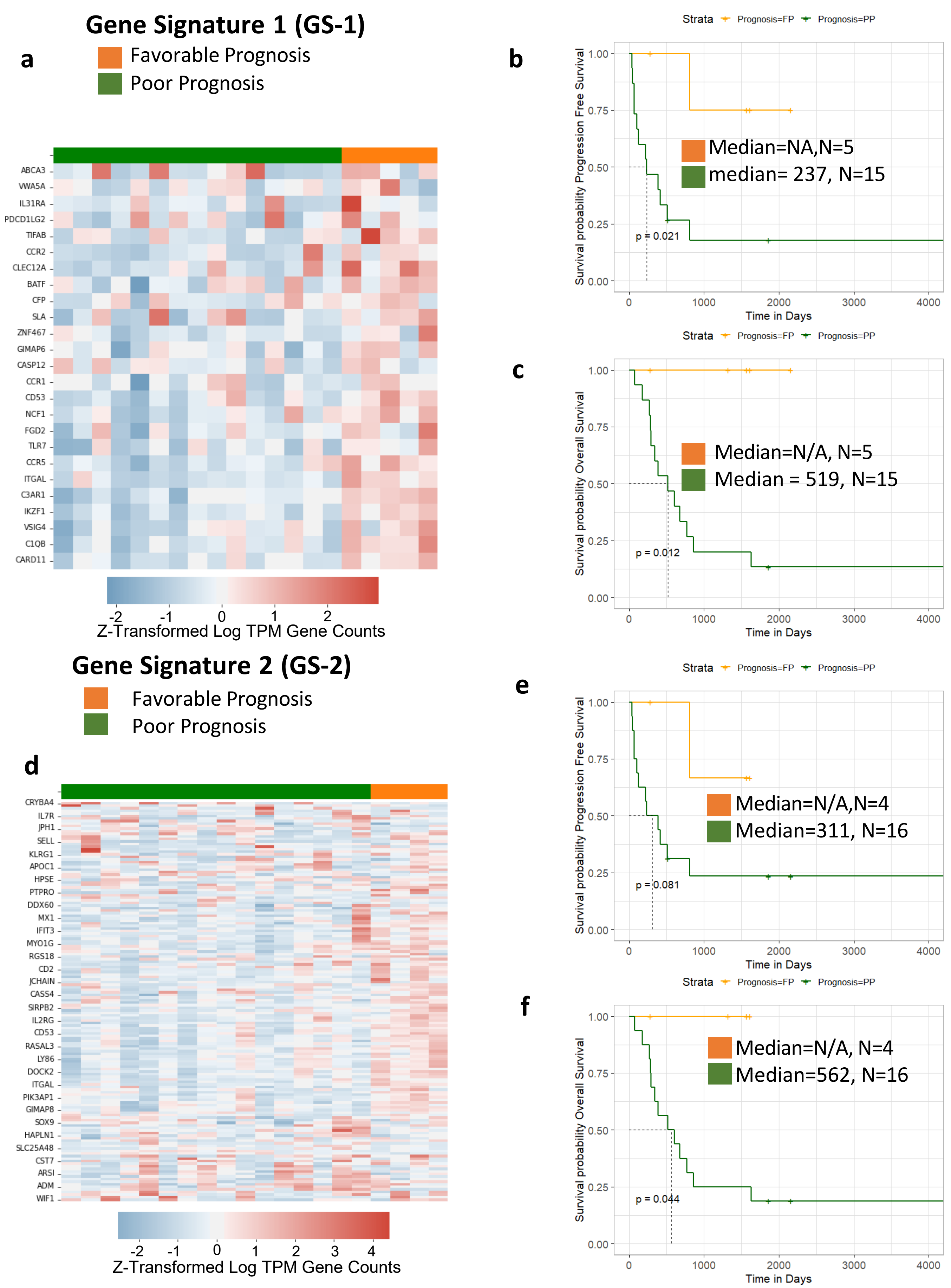

Supplementary Figure 2. Canine osteosarcoma-derived gene signatures are predictive in TARGET metastatic patients. A small number of samples cluster under GS-1 **a** and show significantly different Kaplan-Meier curves for progression free survival (PFS) **b** and overall survival (OSv) **c**. For GS-2 the clustering is not as apparent **d** and the Kaplan-Meier Curves are not significant for PFS **e** but are for OSv **f**. Median PFS and OSv are given in days from diagnosis.

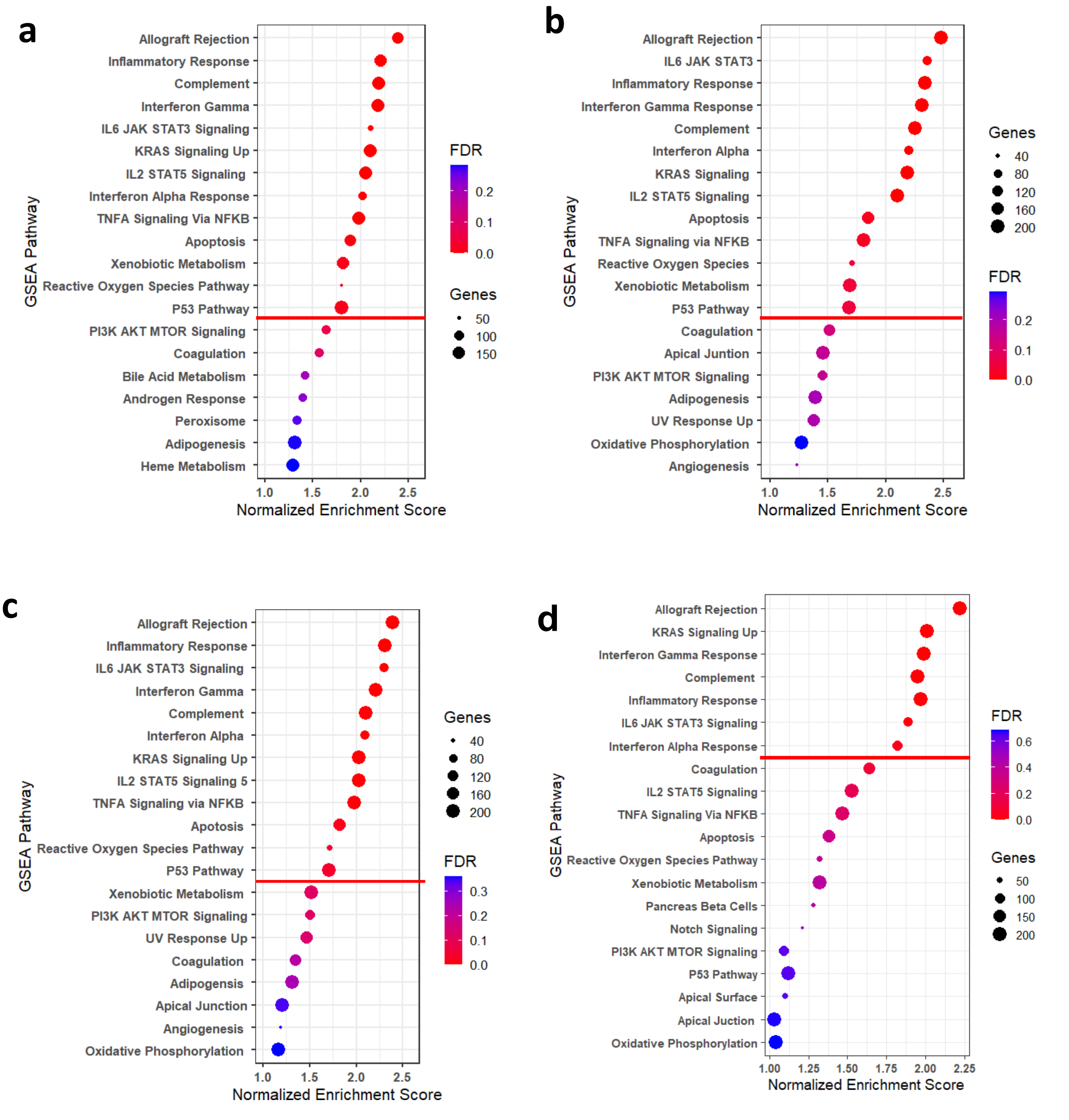

Supplementary Figure 3. Transcriptionally-defined clusters are enriched for specific cellular processes. Normalized Enrichments Scores for gene set enrichment analysis (GSEA) of the top 20 pathways over-represented in the favorable response group when compared to the poor response group when clustered by GS-2 in **a.** DOG[2] **b**. TARGET, **c**. TARGET Non-metastatic patients. **d**. TARGET Metastatic patients.
T Dot size is representative of the number of genes in the pathway and color is indicative of FDR-q value calculated by software, the red line indicates the significance cutoff.

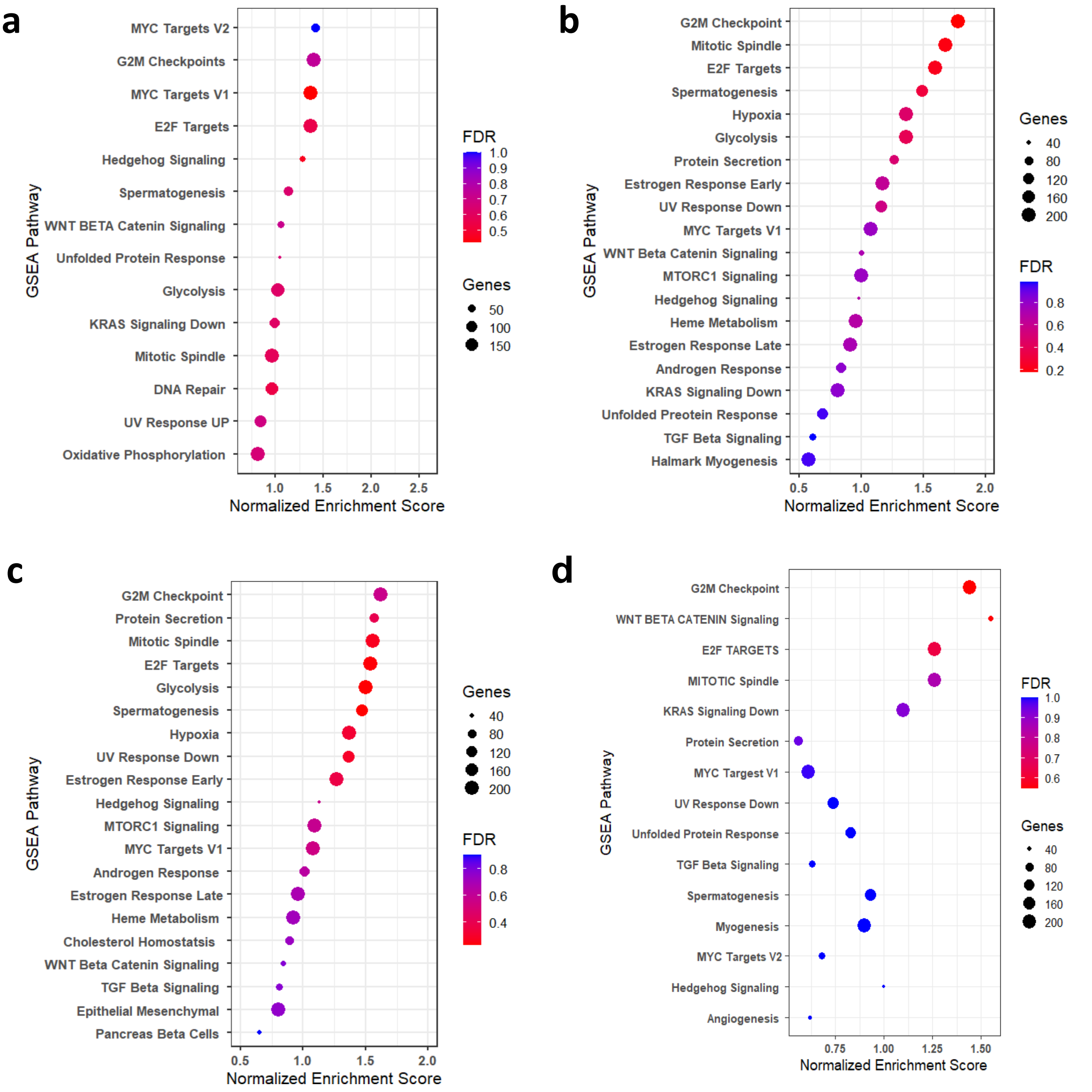

Supplementary Figure 4. No pathways are significantly enriched in the poor prognosis group in GS-1. Listed are pathways GSEA determined to be slightly enriched in the poor prognostic group, but none meet significance criteria. **a.** DOG[2], **b.** TARGET, **c.** TARGET Non-metastatic, **d**. TARGET Metastatic

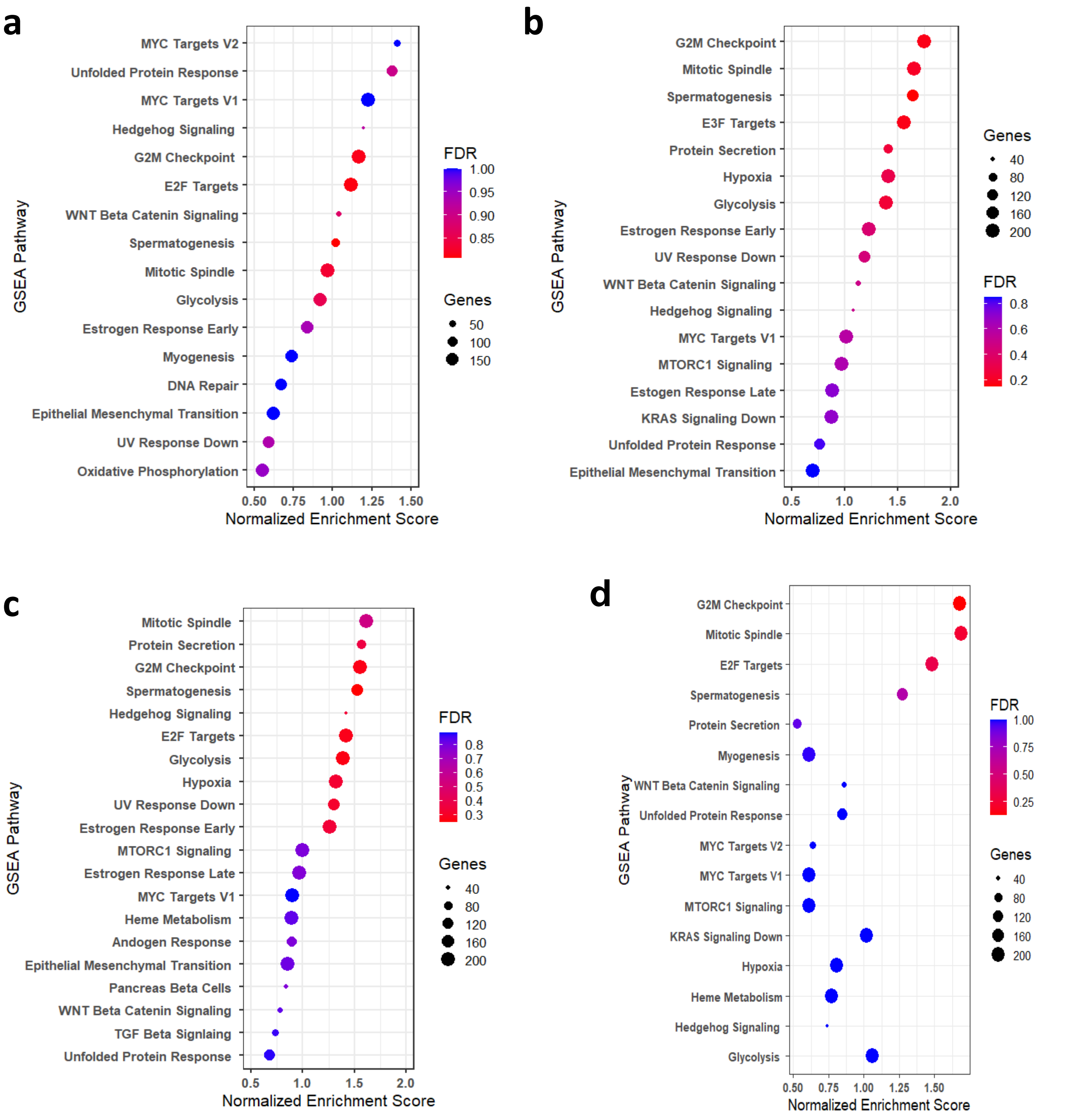

Supplementary Figure 5. No pathways are significantly enriched in the poor prognosis group in GS-2. Listed are pathways GSEA determined to be slightly enriched in the poor prognostic group, but none meet significance criteria. **a**. DOG^2, **b**. TARGET **c**. TARGET Non-metastatic patients. **d**. TARGET Metastatic patients.

a

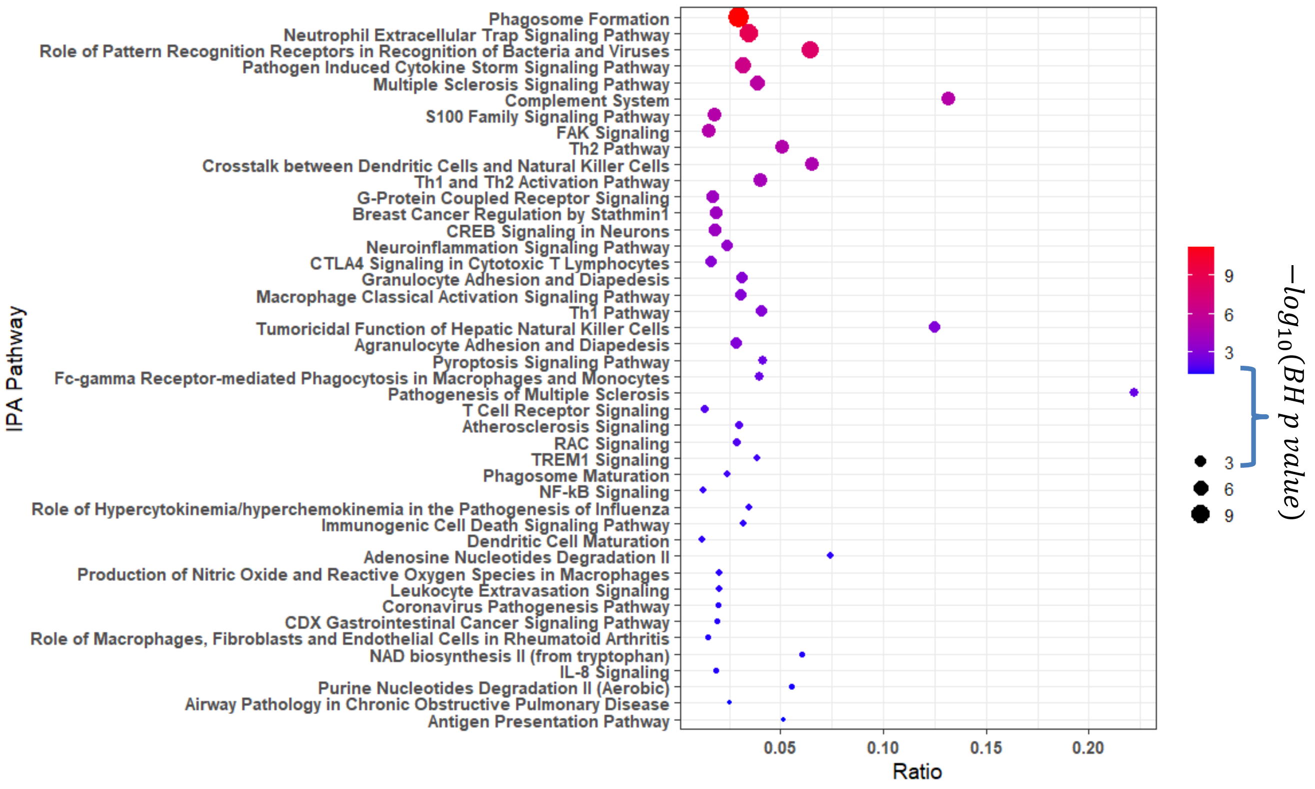

b

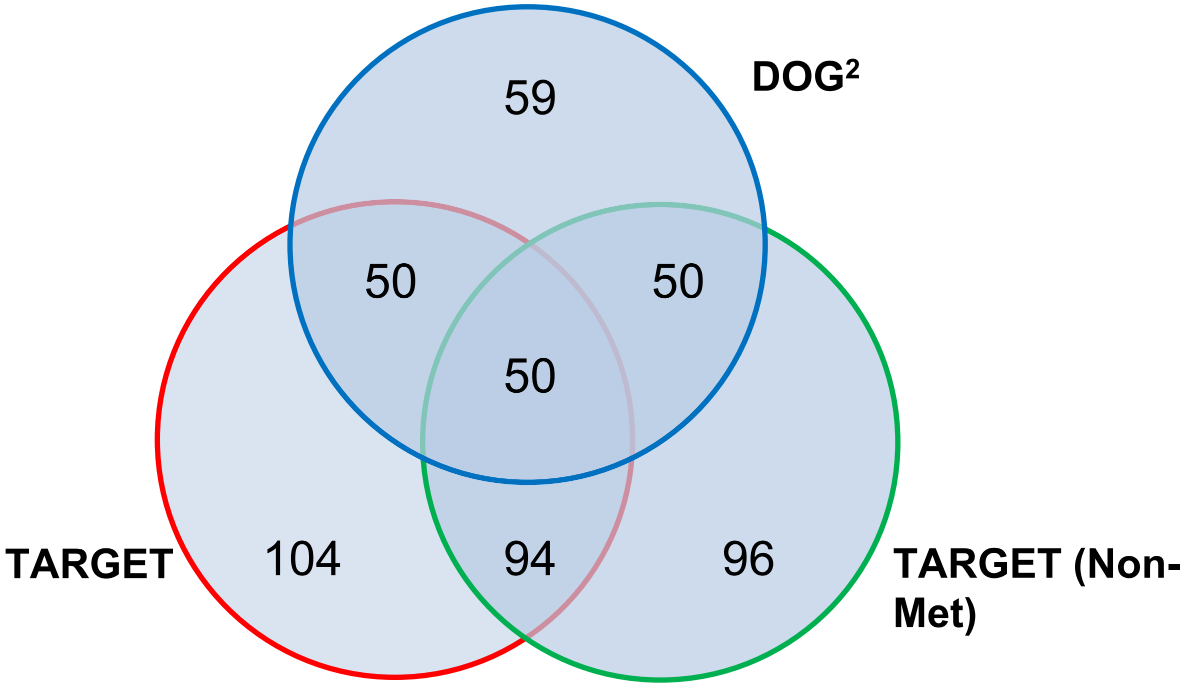

Supplementary Figure 6. Differential Expression Analysis Points to Overlapping Pathways in Canine and Human Osteosarcoma. a. Significant cellular pathways, having a BH corrected p-value less than 0.05, ($-log_{10}(BH\ p\ value)$>1.3), that resulted from IPA analysis on differentially expressed genes that were common to DOG^2, TARGET, and TARGET Non-Metastatic datasets. **b**. The number of significant IPA pathways, as described above, when analysis is conducted on DEGs from each dataset individually.

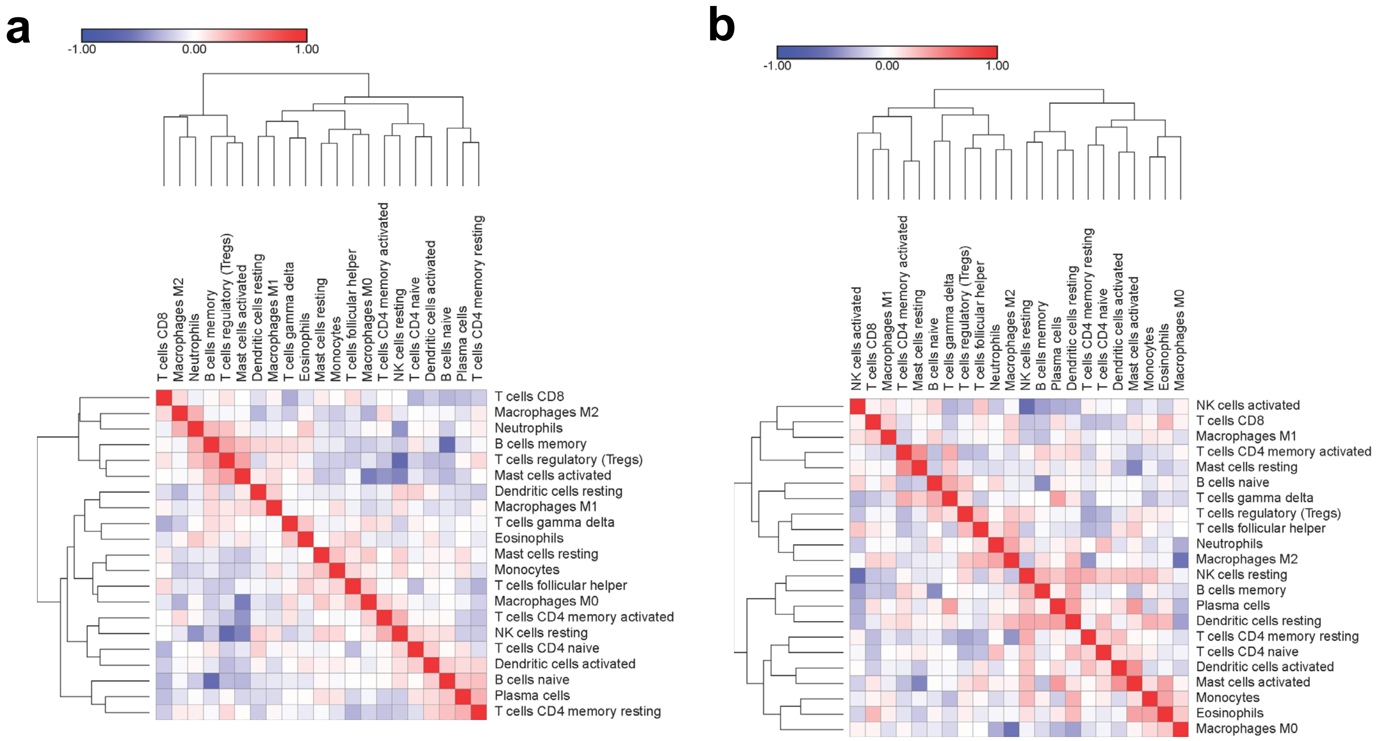

Supplementary Figure 7. Spearman Correlation between CIBERSORTx scores for the PP group for **a**. DOG^2 and **b**. TARGET

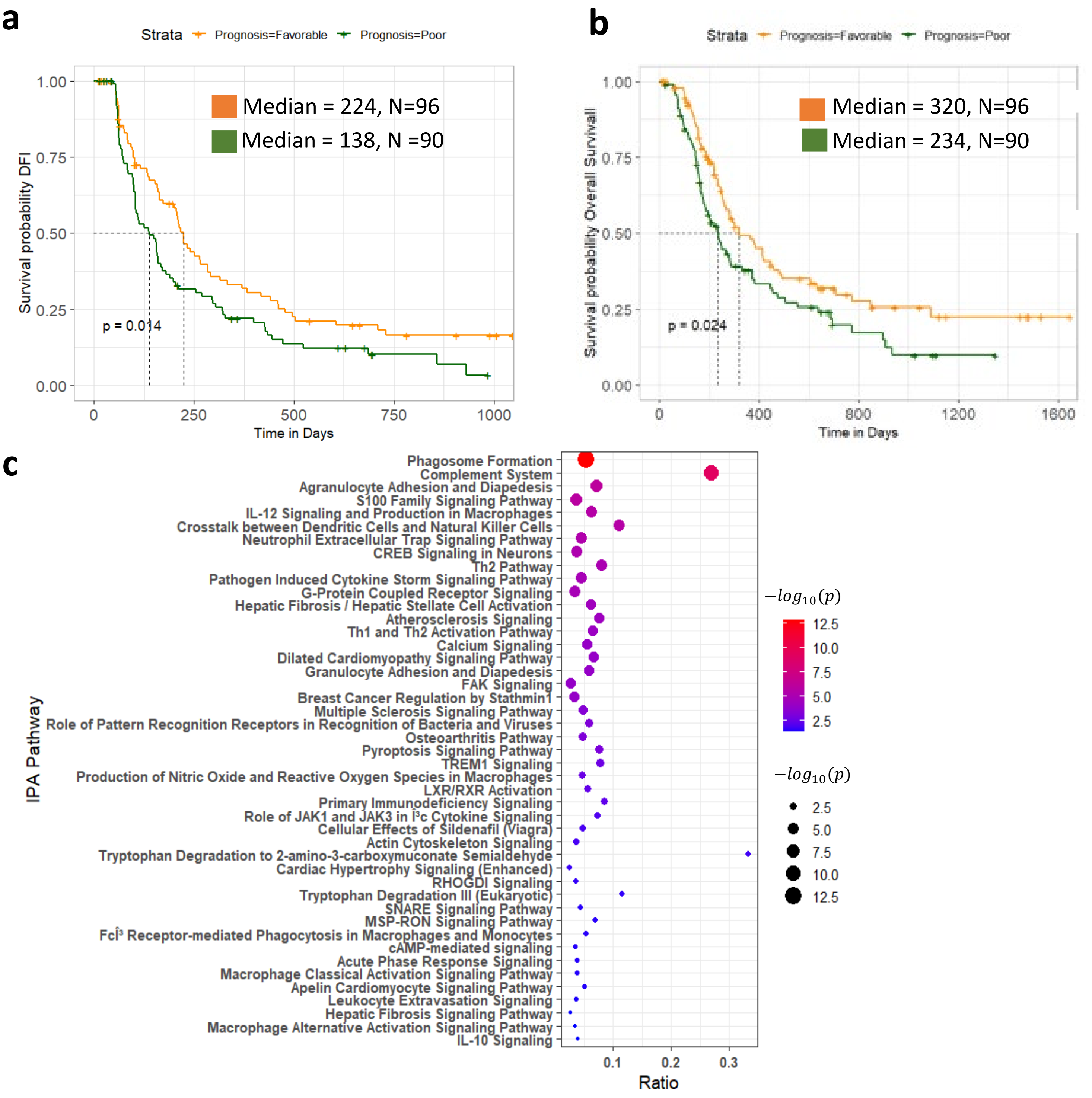

Supplementary Figure 8. Kaplan-Meier curves for DOG^{2} patients formed by K-means clustering on ssGSEA scores for mSigDB hallmark pathways for **a**. Disease Free Interval and **b**.Overall Survival. Significant IPA pathways **c**. associated with DEGs associated with ssGSEA clusters p values are Bonferroni Corrected, Median values for DFI and OSv are given in days from diagnosis.

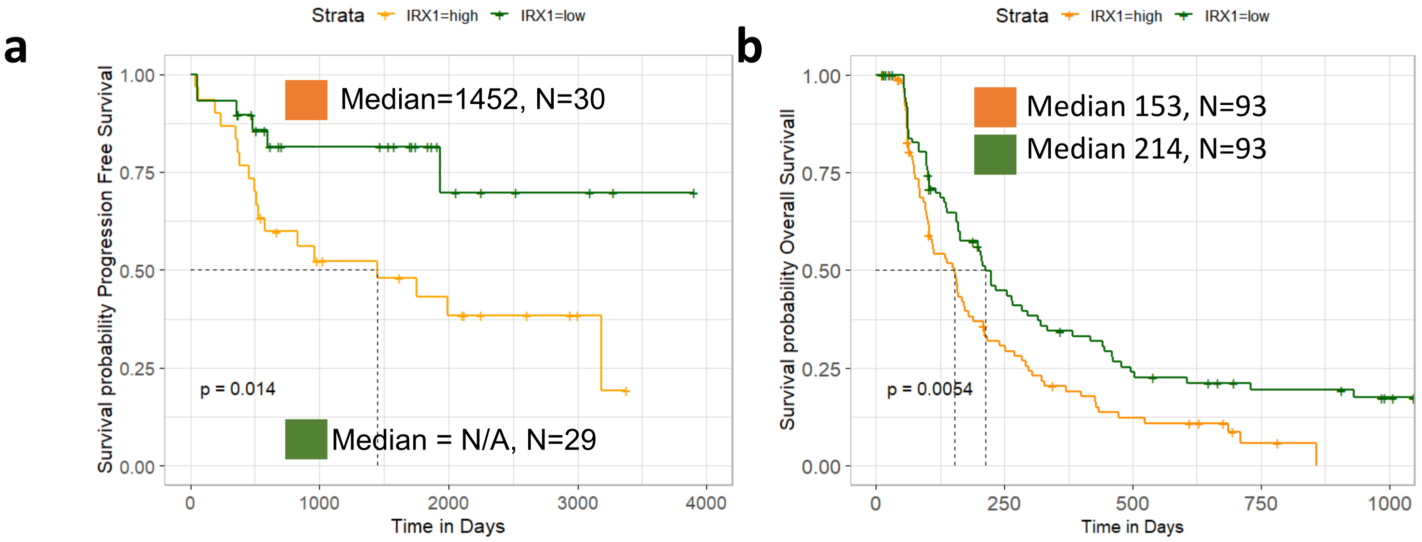

Supplementary Figure 9. Kaplan-Meier curves corresponding to high and low expression of IRX1 and impact on **a** progression-free survival in Non-Metastatic TARGET patients and **b** disease free interval for DOG[2] patients.

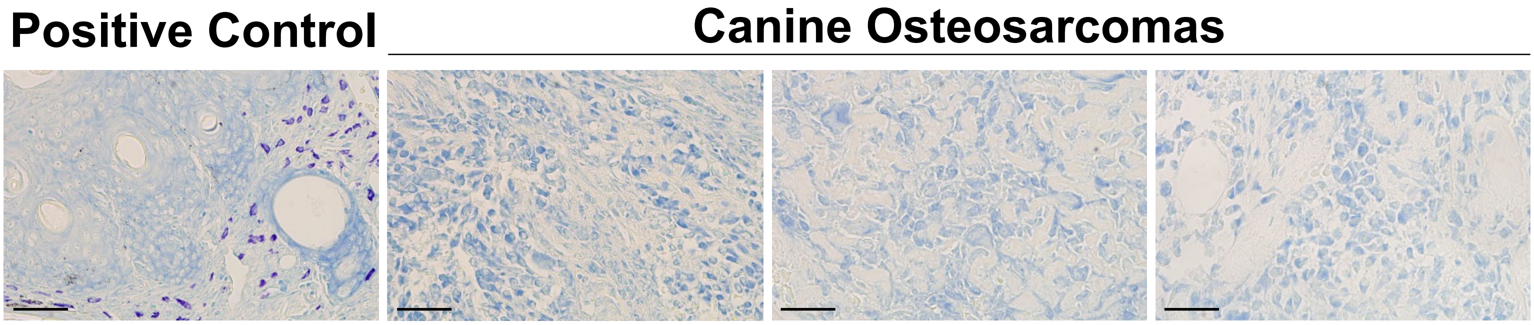

Supplementary Figure 10: Representative images of toluidine blue staining in three canine osteosarcomas. Cytoplasmic granules within mast cells are stained purple in the control tissue (murine skin). Scale bar = 50 µm

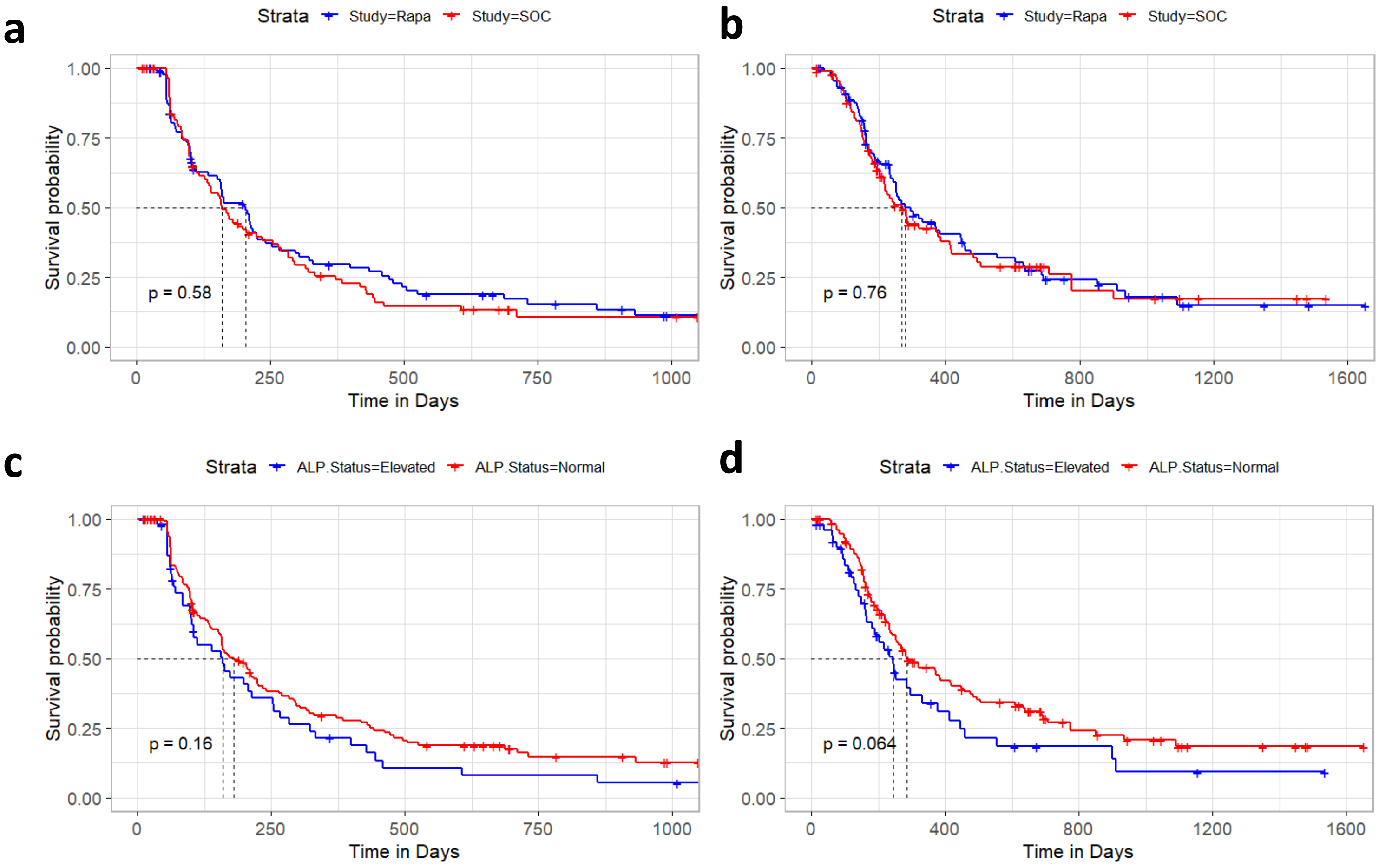

Supplementary Figure 11. Kaplan-Meier Analysis for n = 186 dogs with appendicular osteosarcoma treated with either Standard of Care (SOC, red line) or SOC + adjuvant rapamycin therapy (Rapa, blue line) **a.** Disease-Free Interval (DFI) comparison between SOC (Median DFI of 160 days) vs. SOC+ rapamycin (Median DFI 160 days). **b.** Overall survival comparison (Median SOC survival: 269 days vs. SOC + Rapa 280 days). **c.** DFI comparison between dogs with elevated (blue line) vs. normal ALP levels (red line); median DFI (elevated: 160 days, Normal: 180 days). **d.** Overall survival comparison based on ALP status (median survival elevated ALP: 245 days vs. Normal ALP: 284 days)

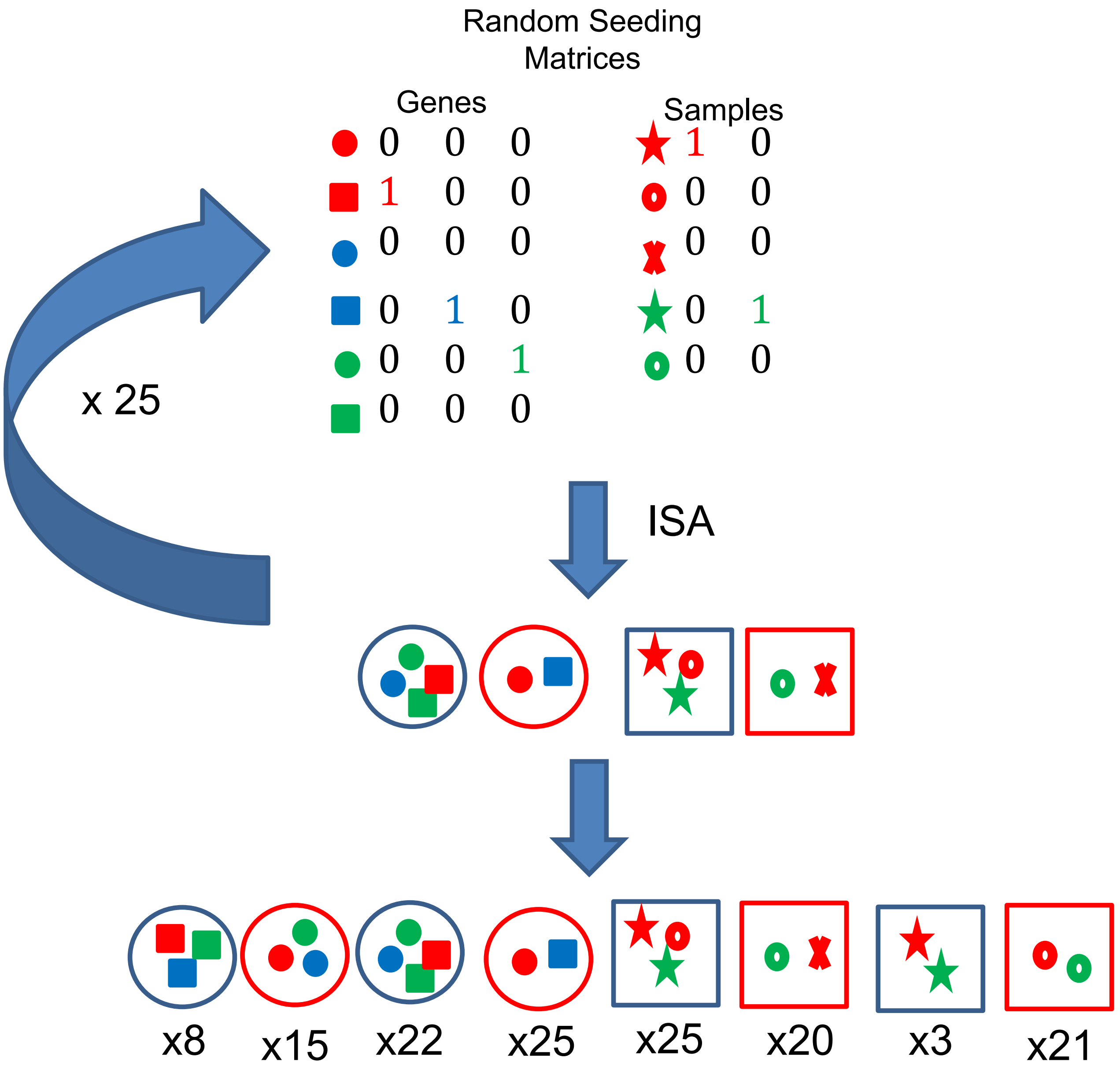

Supplementary Figure 12: Schematic of how random seeding mechanism and robust signature generation was performed. Genes and samples were initially clustered using hierarchical clustering represented by the color above (shapes represent individual genes/samples). Random seeding matrices are constructed by choosing a number of random genes/samples from each initial cluster to initialize ISA. ISA is performed bi-clusters are formed for genes and samples. This process is repeated 25 times forming a distribution of bi-clusters. Only bi-clusters that occur at least in 75% of the trials are kept for further application to gene sets.

Supplementary Note 1
Custom Written Code for Processing and Analyzing Data

*Note All Scripts are written in Python or R and are freely available

TMM_Normaliztion.R: Purpose to filter out low count genes in DOG^2 data

```
setwd("cwd")

#This script takes in raw RNA SEQ reads Performs TMM Normalization

#and remove low count reads. Then corrects for batch effects. This
data was

#ultimately used to filter out low count reads in TPM normalized data
that was

#used in several analysis

library(sva)

library(edgeR)

Xdata<-
read.csv(file='Raw_RNA_SEQ_For_Normaliztion.csv',header=TRUE,row.names
=1)

mdata<-
read.csv(file='Batch_IDs_For_Normalization.csv',header=TRUE,row.names=
1)

batch_labels<-as.factor(mdata$Batch.ID)# Batch labels correspond to
the two

# trials that DOG2 came from

Xcount<-DGEList(Xdata)

keep<-filterByExpr(Xcount,min.count=10,min.prop=0.5,large.n=5)# Filter
out low

# expression

Xv<-Xcount[keep,,keep.lib.sizes=FALSE]

calcNormFactors(Xv, method='TMM') # TMM Normalization

Xcpm<-cpm(Xv,normalized.lib.sizes=TRUE,log = TRUE)

Xcombat<-ComBat(Xcpm,batch=batch_labels) # Batch Correction

write.csv(Xcombat,file="Canine_TMM_Combat_Log_CPM.csv")
```

km_clust.py: K Means Clustering by gene signatures and alignment with Prognostic Data Used. Format the same for canine and human. Same format was used for k-means of clustering ssGSEA

```
import numpy as np

import pandas as pd

from sklearn.preprocessing import StandardScaler

from sklearn.cluster import KMeans

from sklearn.metrics import pairwise_distances

from sklearn import metrics

import matplotlib.pyplot as plt

import sys

import pdb

import os


wd=os.getcwd()


f1=RNA_SEQ_FILE.csv

#Note this is TPM not TMM Data

f2='Clinical_data.csv'


# Align and RNA and Survival Data

DF1=pd.read_csv(f1,delimiter=',',index_col=0)

DF2=pd.read_csv(f2,delimiter=',',index_col=0)


genes1=np.array(DF1.columns)

pids1=np.array(DF1.index)

Xd=np.array(DF1.iloc[:,:])


pids2=np.array(DF2.index)

DFI=np.array(DF2['DFI '])

censor=np.array(DF2['DFI.Censor'])
```

```
indx1=np.arange(pids1.size)[np.in1d(pids1,pids2)]
npids1=pids1[indx1]
Xd=Xd[indx1,:]

idx1=np.argsort(npids1)
npids1=npids1[idx1]
Xd=Xd[idx1,:]

indx2=np.arange(pids2.size)[np.in1d(pids2,npids1)]
npids2=pids2[indx2]
DFI=DFI[indx2]
censor=censor[indx2]

idx2=np.argsort(npids2)
npids2=npids2[idx2]
DFI=DFI[idx2]
censor=censor[idx2]

if np.array_equal(npids1,npids2)==False:
    print("Samples Not Aligned!")
    sys.exit()

# Standardize Data
Xd=np.log2(Xd+2)
ss=StandardScaler()
Xd=ss.fit_transform(Xd)

symbols=[]
for i in range(genes1.size):
    s1=genes1[i]
    symbols.append(s1[:-2])
```

```
symbols=np.array(symbols)

# Select out gene signature genes
f3='Gardner_GS_1_genes.csv'
DF3=pd.read_csv(f3,delimiter=',',index_col=0)
pgenes=np.array(DF3.iloc[:,0])

indx3=np.arange(symbols.size)[np.in1d(symbols,pgenes)]
ngenes=symbols[indx3]
Xn=Xd[:,indx3]

ridx=np.random.choice(np.arange(Xn.shape[0]),Xn.shape[0],replace=False
)
Xn=Xn[ridx,:]
DFI=DFI[ridx]
npids1=npids1[ridx]
censor=censor[ridx]

#Kmeans Cluster
kmv=[2,3,4,5,6]
sil_score=[]
cl_dist=[]
for k in kmv:
    kmeans=KMeans(n_clusters=k)
    kmeans.fit(Xn)
    clust=kmeans.labels_

sil_score.append(metrics.silhouette_score(Xn,clust,metric='euclidean')
)

sil_score=np.array(sil_score)
idxk=np.argmax(sil_score)[0]
```

```
km=KMeans(n_clusters=kmv[idxk])

km.fit(Xn)

labels=km.labels_


dout=np.transpose(np.vstack((DFI,censor,labels)))

dfout=pd.DataFrame(dout,index=npids1,columns=['DFI','DFI.Censor','Clus
ter'])

dfout.to_csv('Gene_Signature_k_means_clustered_data_out.csv')
```

get_cmap.py: Script for creating plots in figures 2,3,7,8,9

```
import numpy as np
import pandas as pd
from sklearn.preprocessing import StandardScaler
import matplotlib.pyplot as plt
import seaborn as sns
import sys

#This script was used to create heatmaps in figure 2,3,7,8,9
f1='RNA_SEQ_Expression_Data.csv'
f2='Clinical_data.csv'

#Load, Allign, and standardize Data
DF1=pd.read_csv(f1,delimiter=',',index_col=0)
DF2=pd.read_csv(f2,delimiter=',',index_col=0)

pids1=np.array(DF1.index)
genes=np.array(DF1.columns)
Xd=np.array(DF1.iloc[:,:])

Xd=np.log2(Xd+1)

ss=StandardScaler()
Xd=ss.fit_transform(Xd)

pids2=np.array(DF2.index)
cid=np.array(DF2['Cluster'])

indx1=np.arange(pids1.size)[np.in1d(pids1,pids2)]
npids1=pids1[indx1]
```

```
Xd=Xd[indx1,:]

idx1=np.argsort(npids1)
npids1=npids1[idx1]
Xd=Xd[idx1,:]

indx2=np.arange(pids2.size)[np.in1d(pids2,npids1)]
npids2=pids2[indx2]
ncid=cid[indx2]

idx2=np.argsort(npids2)
npids2=npids2[idx2]
ncid=ncid[idx2]

if np.array_equal(npids1,npids2)==False:
    print("Labels not Aligned")
    sys.exit()



f3='Gardner_Immuno_Genes_Jan_4_2022.csv'
DF3=pd.read_csv(f3,delimiter=',',index_col=0)
pgenes=np.array(DF3.iloc[:,:])



indx3=np.arange(genes.size)[np.in1d(genes,pgenes)]
ngenes=genes[indx3]
Xn=Xd[:,indx3]



#Cluster genes for better heatmaps
dfplot=pd.DataFrame(Xn,index=npids1,columns=ngenes)
```

```
g=sns.clustermap(dfplot,center=0,cmap=sns.diverging_palette(240, 15,
as_cmap=True),yticklabels=False,cbar_pos=(1,0.2,0.05,0.5))

idxc=g.dendrogram_col.reordered_ind
Xn=Xn[:,idxc]
Ngenes=ngenes[idxc]

idxA=np.where(ncid==1)[0]
idxB=np.where(ncid==0)[0]

XnA=Xn[idxA,:]
XnB=Xn[idxB,:]
npidsA=npids1[idxA]
npidsB=npids1[idxB]

XN=np.vstack((XnA,XnB))
Npids=np.append(npidsA,npidsB)
labels=np.zeros((Npids.size,),dtype=int)
labels[:npidsA.size]=1

# Make final heatmap
dfplot2=pd.DataFrame(XN,index=Npids,columns=Ngenes)
labels1=pd.Series(labels,index=Npids)
lut1={1:'tab:orange',0:'g'}
row_colors=labels1.map(lut1)
g5=sns.clustermap(dfplot2,center=0,method='average',row_colors=row_col
ors,col_cluster=False,row_cluster=False,cmap=sns.diverging_palette(240
, 15, as_cmap=True),yticklabels=False,cbar_pos=(1,0.2,0.05,0.5))
```

survival_plot.R: Creates Kaplan-Meier Plots and Statistics for all plots.

```
setwd("cwd")
#The Script Calculates and Plots Kaplan Meier Plots in all figures
library("survival")
library("survminer")
Xdata=read.csv(file='Cluster_Survival_Data.csv',sep=',',row.names=1)
fit<-survfit(Surv(PFS,PFS.Censor) ~ Cluster, data=Xdata)
ggsurvplot(fit,pval=TRUE,conf.int=FALSE,surv.median.line = "hv",
           ggtheme = theme_light(16),palette=c("orange","dark green"),
           xlab="Time in Days",ylab='Survival probability Progresion
Free
           Survival',font.x=c(16),font.y=c(16),font.tickslab=c(14))
#calculate median of survival curves
sm=surv_median(fit)
```

GSEA_Format.py: Formats data necessary for GSEA analysis in Figure 3.

```
import numpy as np

import pandas as pd

from sklearn.preprocessing import StandardScaler

from sklearn.cluster import KMeans

from sklearn.metrics import pairwise_distances

from sklearn import metrics

import sys

import pdb


#This Formats Cluster labeled data and expression data for GSEA analysis

#presented in figure  4


f1='Expression_Matrix.csv'

DF1=pd.read_csv(f1,delimiter=',',index_col=0)

pids1=np.array(DF1.index)

genes1=np.array(DF1.columns)

Xd=np.array(DF1.iloc[:,:])



ngenes=genes1


f2='Cluster_Labels_as_Data.csv'

DF2=pd.read_csv(f2,delimiter=',',index_col=0)

pids2=np.array(DF2.index)

clid=np.array(DF2['Cluster'])


indx1=np.arange(pids1.size)[np.in1d(pids1,pids2)]

npids1=pids1[indx1]
```

```
Xd=Xd[indx1,:]

idx1=np.argsort(npids1)
npids1=npids1[idx1]
Xd=Xd[idx1,:]

indx2=np.arange(pids2.size)[np.in1d(pids2,npids1)]
npids2=pids2[indx2]
nclid=clid[indx2]

idx2=np.argsort(npids2)
npids2=npids2[idx2]
nclid=nclid[idx2]

if np.array_equal(npids1,npids2)==False:
    print("PIDS Not Aligned")
    sys.exit()

Xd=np.log2(1+Xd)
ss=StandardScaler()
Xd=ss.fit_transform(Xd)

idxB=np.where(nclid==0)[0]
idxA=np.where(nclid==1)[0]

labels=np.zeros((nclid.size,),dtype=str)
labels[idxA]='P'
labels[idxB]='F'

Xn=np.transpose(Xd)
dfout1=pd.DataFrame(Xn,index=ngenes,columns=npids1)
```

```
dfout2=pd.DataFrame(np.repeat('NA',ngenes.size),index=ngenes,columns=[
'DESCRIPTION'])

dfout=pd.concat([dfout2,dfout1],axis=1)

labels=labels.reshape(1,labels.size)

np.savetxt('GSEA_Labels.cls',labels,fmt='%s',delimiter='\t')

dfout.to_csv('Gardner_GSEA_Expression.csv')
```

Limma_Dog2.py: Performs differential expression via Limma-voom pipeline on DOG^2 data

```
setwd("/Users/mannheimerjd/documents/Transcriptomics_V2/DOG2_V2")

#This script perform differential expression analysis for DOG2 Data

# Note that the Limma Pipeline requires raw RNA SEQ values

# Additionally Batch informtation must be included so it can be
accounted for

# in the linear model.

library(edgeR)

# Input is RNA Read count matrix and a file with batch a k-means
cluster labels

# for a given gene signature. These data files are aligned by patient

#id prior to loading


Xdata1<-
read.csv('Canine_Hallmarks_limma_RNA_Data_TPM.csv',header=TRUE,

                 row.names=1)#RNA Count Data

label_data<-
read.csv('Canine_TPM_Hallmark_Limma_labels.csv',header=TRUE,

                     row.names=1)# Batch and cluster info

batch<-as.factor(label_data$Batch.ID)

cluster=as.factor(label_data$Cluster)

Xd<-DGEList(Xdata1)

keep<-filterByExpr(Xd,min_count=10,min.prop=0.5,large.n=5)

X0<-Xd[keep,, keep.lib.sizes=FALSE]

X0<-calcNormFactors(X0,method='TMM') #Normalization

mm<-model.matrix(~batch+cluster)# Linear model is both cluster and
batch

y<-voom(X0,mm,plot=T)

fit1<-lmFit(y,mm)

fit<-eBayes(fit1)
```

```
#We identify signficant DEGs based on the linear coefficients
conditioned on

#cluster label for each gene.

tpt.table<-topTable(fit,coef='cluster1',sort.by =
'P',number=Inf,adjust="BH")

adjp<-tpt.table$adj.P.Val

Ngenes<-row.names(tpt.table)

idx<-which(adjp<0.05)

dfout<-tpt.table[idx,]

write.csv(dfout,file='Data_Results.csv')
```

Targets_limma.py: Performs Limma-voom analysis on TARGET DATA

```
setwd("cwd")

library(edgeR)

# Performs Limma Voom Differential Expression Analysis. The only

# difference between this and the canine is that there is not term

# for batch correction is there is no batch information for TARGET

# data set

Xdata1<-read.csv('RNA_READ_Counts.csv',header=TRUE,row.names = 1)

DSMAT<-read.csv('Limma_Labels.csv',header = TRUE,row.names=1)

cluster<-as.factor(DSMAT$Cluster)

Xd<-DGEList(Xdata1)

keep<-filterByExpr(Xd,DSMAT,min.count=10,min.prop=0.5,large.n=5)

X0<-Xd[keep,, keep.lib.sizes=FALSE]

X0<-calcNormFactors(X0,method='TMM')

mm<-model.matrix(~cluster)

y<-voom(X0,mm,plot=T)

fit1<-lmFit(y,mm)

fit<-eBayes(fit1)

tpt.table<-topTable(fit,coef='cluster1',sort.by =
'p',number=Inf,adjust="BH")

adjp<-tpt.table$adj.P.Val

Ngenes<-row.names(tpt.table)

idx<-which(adjp<0.05)

dfout<-tpt.table[idx,]

write.csv(dfout,file='TARGET_Limma_DGES.csv')
```

get_ran_clust_seeds.py: Randomly select seeds for ISA algorithm.

```
import numpy as np

import pandas as pd

from sklearn.preprocessing import StandardScaler

import sys


# This script generates the seed matrices necessary for the ISA
alogorithm

# The alogoritm requires seed matrices for both genes and samples


f1='Canine_DEG_RNA_SEQ_Data.csv' # log and normed RNA data of genes
derived

# from DEG analysis on ssGSEA kmeans cluster

f2='Hclust_for_Seeds.csv' # Intial Clustering obtained by heirarchial

#clustering of genes. This was done using hclust in R and cutree in r
this

# was done for n=10

f3='Hclust_7_features_for_seeds.csv' # Clusters for patients obtained
the

# same way as genes with the exception n=7


DF1=pd.read_csv(f1,delimiter=',',index_col=0)

DF2=pd.read_csv(f2,delimiter=',',index_col=0)

DF3=pd.read_csv(f3,delimiter=',',index_col=0)


pids1=np.array(DF1.index)

genes1=np.array(DF1.columns)

Xd=np.array(DF1.iloc[:,:])


genes2=np.array(DF2.index)
```

```
clust1=np.array(DF2.iloc[:,0])

pids2=np.array(DF3.index)
clust2=np.array(DF3.iloc[:,0])


idx1=np.argsort(pids1)
idx2=np.argsort(genes1)
ngenes1=genes1[idx2]
npids1=pids1[idx1]
Xd=Xd[idx1,:]
Xd=Xd[:,idx2]

idx3=np.argsort(genes2)
ngenes2=genes2[idx3]
clust1=clust1[idx3]

idx4=np.argsort(pids2)
npids2=pids2[idx4]
clust2=clust2[idx4]

if np.array_equal(ngenes1,ngenes2)==False:
    print("genes not aligned")
    sys.exit()

if np.array_equal(npids1,npids2)==False:
    print("pids not aligned")

seeds1=np.zeros((ngenes1.size,10),dtype=int)
seeds2=np.zeros((npids1.size,7),dtype=int)

# Randomly select 3 genes from each initial cluster as seeds
```

```
for i in range(10):

    c=i+1

    idxA=np.where(clust1==c)[0]

    ran1=np.random.choice(idxA,3,replace=False)

    seeds1[ran1,i]=1


# Randomly select 3 pids from each intital cluster as seeds

for i in range(7):

    c=i+1

    idxB=np.where(clust2==c)[0]

    ran2=np.random.choice(idxB,3,replace=False)

    seeds2[ran2,i]=1


dfout2=pd.DataFrame(seeds1,index=ngenes2,columns=['1','2','3','4','5',
'6','7','8','9','10'])

dfout3=pd.DataFrame(seeds2,index=npids2,columns=['1','2','3','4','5','
6','7'])


dfout2.to_csv('Gene_Seeds.csv')

dfout3.to_csv('Sample_Seeds.csv')
```

ISA_clustering.R Performs ISA clustering

```
setwd("/Users/mannheimerjd/documents/Transcriptomics_V2/DOG2_V2")

library('eisa')

# This script perfroms the ISA algorithm on RNA data of the DEGs obtained

# the DEGs obtained from k-means and limma analysis on ssGSEA data

# the number of content of clusters is dependent on the random seed matrices

# used to initialize the data, and the cutoff "thr.feat" and "thr.samp" required

# by the algorithm. The output is both gene clusters and sample clusters. This

# was repeated 25 times and clusters of both gene and sample that occured 75%

#were kept.

Xdata<-read.csv(file='RNA_SEQ_DATA.csv',sep=',',row.names=1)

Fseed<-read.csv(file='Gene_Seeds.csv',sep=',',row.names=1)

Sseed<-read.csv(file='Sample_Seeds.csv',sep=',',row.names=1)

Xdata<-t(as.matrix(Xdata))

Xmat<-ExpressionSet(assayData=Xdata)

annotation(Xmat)<-"org.Cf.eg"

fseeds<-as.matrix(Fseed)

sseeds<-as.matrix(Sseed)

Xmat.norm<-ISANormalize(Xmat)

biclust<-ISAIterate(Xmat.norm,feature.seeds=fseeds,sample.seeds = sseeds ,thr.feat=1.5, thr.samp=0.7)

bc.unique<-ISAUnique(Xmat.norm,biclust)

bc.robust<-ISAFilterRobust(Xmat.norm,bc.unique)

fmat<-getFeatureMatrix(bc.robust)

smat<-getSampleMatrix(bc.robust)

write.csv(fmat,file='Feature_Matrix_25.csv')
```

```
write.csv(fmat,file='Sample_Matrix_25.csv')
```
