# Supplementary material for: Transcriptional profiling of canine osteosarcoma identifies prognostic gene expression signatures with translational value for humans
Source: Commun Biol. 2023 Aug 17;6:856. doi: 10.1038/s42003-023-05208-z (PMC10435536; doi:10.1038/s42003-023-05208-z)
Supplement: Supplementary file 3 — Description of Supplementary Materials [file 42003_2023_5208_MOESM3_ESM.docx]

**Description of Additional Supplementary Files**

**File name:** Supplementary Data 1

**Description:** Contains raw data behind all main manuscript and supplementary figures.

**File name:** Supplementary Data 2

**Description:** Contains lists of pathways referenced in IPA, GSEA, DEG analyses presented in the manuscript.
