# Supplementary material for: Transcriptional profiling of canine osteosarcoma identifies prognostic gene expression signatures with translational value for humans
Source: Commun Biol. 2023 Aug 17;6:856. doi: 10.1038/s42003-023-05208-z (PMC10435536; doi:10.1038/s42003-023-05208-z)
Supplement: Supplementary file 6 — Reporting Summary [file 42003_2023_5208_MOESM6_ESM.pdf]

nature portfolio

Corresponding author(s): COMMSBIO_22_4011 Transcriptional profiling of canine osteosarcoma identifies prognostic gene expression signatures with translational value for humans

Last updated by author(s): 2023-7-28

Reporting Summary

Nature Portfolio wishes to improve the reproducibility of the work that we publish. This form provides structure for consistency and transparency in reporting. For further information on Nature Portfolio policies, see our Editorial Policies and the Editorial Policy Checklist.

Statistics

For all statistical analyses, confirm that the following items are present in the figure legend, table legend, main text, or Methods section.

n/a	Confirmed	
☐	☒	The exact sample size (*n*) for each experimental group/condition, given as a discrete number and unit of measurement
☐	☒	A statement on whether measurements were taken from distinct samples or whether the same sample was measured repeatedly
☐	☒	The statistical test(s) used AND whether they are one- or two-sided<br>*Only common tests should be described solely by name; describe more complex techniques in the Methods section.*
☐	☒	A description of all covariates tested
☐	☒	A description of any assumptions or corrections, such as tests of normality and adjustment for multiple comparisons
☐	☒	A full description of the statistical parameters including central tendency (e.g. means) or other basic estimates (e.g. regression coefficient) AND variation (e.g. standard deviation) or associated estimates of uncertainty (e.g. confidence intervals)
☒	☐	For null hypothesis testing, the test statistic (e.g. *F*, *t*, *r*) with confidence intervals, effect sizes, degrees of freedom and *P* value noted<br>*Give P values as exact values whenever suitable.*
☒	☐	For Bayesian analysis, information on the choice of priors and Markov chain Monte Carlo settings
☐	☒	For hierarchical and complex designs, identification of the appropriate level for tests and full reporting of outcomes
☐	☒	Estimates of effect sizes (e.g. Cohen's *d*, Pearson's *r*), indicating how they were calculated

Our web collection on statistics for biologists contains articles on many of the points above.

Software and code

Policy information about availability of computer code

Data collection	Details provided in Methods
Data analysis	Details provided in Methods. Code has been deposited into Zenodo.

For manuscripts utilizing custom algorithms or software that are central to the research but not yet described in published literature, software must be made available to editors and reviewers. We strongly encourage code deposition in a community repository (e.g. GitHub). See the Nature Portfolio guidelines for submitting code & software for further information.

Data

Policy information about availability of data

All manuscripts must include a data availability statement. This statement should provide the following information, where applicable:

- Accession codes, unique identifiers, or web links for publicly available datasets
- A description of any restrictions on data availability
- For clinical datasets or third party data, please ensure that the statement adheres to our policy

Data availability statement:
The datasets generated during and/or analyzed during the current study are available at GEO accession GSE238110

Human research participants

Policy information about studies involving human research participants and Sex and Gender in Research.

Reporting on sex and gender	*Use the terms sex (biological attribute) and gender (shaped by social and cultural circumstances) carefully in order to avoid confusing both terms. Indicate if findings apply to only one sex or gender; describe whether sex and gender were considered in study design whether sex and/or gender was determined based on self-reporting or assigned and methods used. Provide in the source data disaggregated sex and gender data where this information has been collected, and consent has been obtained for sharing of individual-level data; provide overall numbers in this Reporting Summary. Please state if this information has not been collected. Report sex- and gender-based analyses where performed, justify reasons for lack of sex- and gender-based analysis.*
Population characteristics	*Describe the covariate-relevant population characteristics of the human research participants (e.g. age, genotypic information, past and current diagnosis and treatment categories). If you filled out the behavioural & social sciences study design questions and have nothing to add here, write "See above."*
Recruitment	*Describe how participants were recruited. Outline any potential self-selection bias or other biases that may be present and how these are likely to impact results.*
Ethics oversight	*Identify the organization(s) that approved the study protocol.*

Note that full information on the approval of the study protocol must also be provided in the manuscript.

Field-specific reporting

Please select the one below that is the best fit for your research. If you are not sure, read the appropriate sections before making your selection.

☒ Life sciences ☐ Behavioural & social sciences ☐ Ecological, evolutionary & environmental sciences

For a reference copy of the document with all sections, see nature.com/documents/nr-reporting-summary-flat.pdf

Life sciences study design

All studies must disclose on these points even when the disclosure is negative.

Sample size	Data was generated from privately-owned pet dogs enrolled in a prospective, randomized comparative oncology veterinary clinical trial
Data exclusions	Data was generated from privately-owned pet dogs enrolled in a prospective, randomized comparative oncology veterinary clinical trial. Data was collected as per trial protocol. See LeBlanc AK, Mazcko CN, Cherukuri A, Berger EP, Kisseberth WC, Brown ME, Lana SE, Weishaar K, Flesner BK, Bryan JN, Vail DM, Burton JH, Willcox JL, Mutsaers AJ, Woods JP, Northrup NC, Saba C, Curran KM, Leeper H, Wilson-Robles H, Wustefeld-Janssens BG, Lindley S, Smith AN, Dervisis N, Klahn S, Higginbotham ML, Wouda RM, Krick E, Mahoney JA, London CA, Barber LG, Balkman CE, McCleary-Wheeler AL, Suter SE, Martin O, Borgatti A, Burgess K, Childress MO, Fidel JL, Allstadt SD, Gustafson DL, Selmic LE, Khanna C, Fan TM. Adjuvant Sirolimus Does Not Improve Outcome in Pet Dogs Receiving Standard-of-Care Therapy for Appendicular Osteosarcoma: A Prospective, Randomized Trial of 324 Dogs. Clin Cancer Res. 2021 Jun 1;27(11):3005-3016. doi: 10.1158/1078-0432.CCR-21-0315. Epub 2021 Mar 22. PMID: 33753454; PMCID: PMC8172450.
Replication	N/A
Randomization	N/A
Blinding	N/A

Reporting for specific materials, systems and methods

We require information from authors about some types of materials, experimental systems and methods used in many studies. Here, indicate whether each material, system or method listed is relevant to your study. If you are not sure if a list item applies to your research, read the appropriate section before selecting a response.

Materials & experimental systems

n/a	Involved in the study	
☐	☒	Antibodies
☒	☐	Eukaryotic cell lines
☒	☐	Palaeontology and archaeology
☐	☒	Animals and other organisms
☒	☐	Clinical data
☒	☐	Dual use research of concern

Methods

n/a	Involved in the study	
☒	☐	ChIP-seq
☒	☐	Flow cytometry
☒	☐	MRI-based neuroimaging

Antibodies

Antibodies used	Provided in Supplementary materials
Validation	Provided in methods

Animals and other research organisms

Policy information about studies involving animals; ARRIVE guidelines recommended for reporting animal research, and Sex and Gender in Research

Laboratory animals	N/A
Wild animals	N/A
Reporting on sex	N/A
Field-collected samples	Data was generated from privately-owned pet dogs enrolled in a prospective, randomized comparative oncology veterinary clinical trial
Ethics oversight	All samples analyzed in this manuscript were collected from privately-owned pet dogs enrolled in a clinical trial with IACUC approval. See LeBlanc AK, Mazcko CN, Cherukuri A, Berger EP, Kisseberth WC, Brown ME, Lana SE, Weishaar K, Flesner BK, Bryan JN, Vail DM, Burton JH, Willcox JL, Mutsaers AJ, Woods JP, Northrup NC, Saba C, Curran KM, Leeper H, Wilson-Robles H, Wustefeld-Janssens BG, Lindley S, Smith AN, Dervisis N, Klahn S, Higginbotham ML, Wouda RM, Krick E, Mahoney JA, London CA, Barber LG, Balkman CE, McCleary-Wheeler AL, Suter SE, Martin O, Borgatti A, Burgess K, Childress MO, Fidel JL, Allstadt SD, Gustafson DL, Selmic LE, Khanna C, Fan TM. Adjuvant Sirolimus Does Not Improve Outcome in Pet Dogs Receiving Standard-of-Care Therapy for Appendicular Osteosarcoma: A Prospective, Randomized Trial of 324 Dogs. Clin Cancer Res. 2021 Jun 1;27(11):3005-3016. doi: 10.1158/1078-0432.CCR-21-0315. Epub 2021 Mar 22. PMID: 33753454; PMCID: PMC8172450.

Note that full information on the approval of the study protocol must also be provided in the manuscript.
